# Demographic predictors of major depressive disorder among patients with iron deficiency anemia: a retrospective cross-sectional observational study

**DOI:** 10.1186/s12888-026-08389-z

**Published:** 2026-07-21

**Authors:** Crystal Yu, Christian R. Hardoy, Maria T. Paulino, Ellee P. Vikram, Jacob F. Giffin, Anna S. Aledia, Rimal Bera, Alpesh N. Amin

**Affiliations:** 1 University of California, Irvine School of Medicine, Irvine, CA USA; 2 Department of Psychiatry, University of Arizona, Tucson, AZ USA; 3 Department of Anesthesiology, Brooke Army Medical Center, San Antonio, TX USA; 4 Department of Medicine, Division of Hospital Medicine and Palliative Medicine, University of California, Irvine, USA; 5 Department of Psychiatry and Human Behavior, University of California, Irvine, Orange, CA USA

**Keywords:** Iron deficiency anemia, Major depressive disorder, Demographic disparities

## Abstract

**Background:**

Iron deficiency anemia (IDA) has been increasingly associated with neuropsychiatric conditions, including major depressive disorder (MDD). However, demographic variation in documented MDD diagnoses among patients with IDA remains underexplored in large clinical populations.

**Methods:**

We conducted a retrospective cross-sectional observational study using de-identified electronic health records from 8,593 adult patients at the University of California, Irvine Medical Center (2017–2024). IDA was identified using ICD-10-CM codes, and MDD was classified as single-episode or recurrent. Multivariate logistic regression adjusted for age, sex, race, and ethnicity was used to assess demographic variation in MDD diagnoses among patients with IDA.

**Results:**

Among 8,593 patients with IDA, single-episode MDD was present in 23.2% and recurrent MDD in 7.2%. Female patients demonstrated significantly higher odds of single-episode MDD (OR 1.52, 95% CI 1.35–1.72) and recurrent MDD (OR 1.84, 95% CI 1.50–2.28) compared with males. Adults ≥ 55 years demonstrated increased odds of single-episode MDD (OR 1.62, 95% CI 1.27–2.08 and OR 1.40, 95% CI 1.12–1.76, respectively), with no significant association observed for recurrent MDD. Asian individuals had significantly lower odds of single-episode (OR 0.44, 95% CI 0.37–0.52) and recurrent MDD (OR 0.39, 95% CI 0.28–0.52) than White patients.

**Conclusion:**

This large cross-sectional study identified significant demographic variation in documented MDD diagnoses among patients with IDA. These findings help characterize patterns of psychiatric comorbidity within IDA populations and motivate future longitudinal studies examining the relationship between IDA and depressive disorders.

## Introduction

Anemia is a multifactorial, global health concern in which the body lacks the red blood cells or hemoglobin to adequately supply oxygen [1]. In particular, women and children younger than five years old are significantly more affected by anemia [1–3]. A cross-sectional survey of the United States population conducted between 2021 and 2023 observed a significantly higher prevalence of anemia in females (13.0% v. 5.5%) compared to males [1]. Existing demographic differences in the prevalence of anemia remain an area of interest given the potential negative health outcomes and preventability of the condition. Iron deficiency anemia (IDA) is the most common anemia, affecting 1.2 billion people across the world [1, 3]. The World Health Organization defines IDA as anemia, diagnosed via low hemoglobin concentration, with biochemical evidence of an iron deficiency via low serum ferritin [4]. Iron is known to be a critical contributor to many metabolic processes, including but not limited to immune response, cellular transport, and DNA synthesis [5, 6].

In the nervous system, iron is necessary for proper development via neurotransmitter synthesis, myelination of neurons, and cellular respiration to support biochemical processes that preserve essential cell functions and neural activity [7, 8]. Iron deficiency in pregnancy or childhood has been observed to produce deficits in language comprehension, speech production, memory, and motor skills [8, 9]. Further studies have identified a potential association between iron deficiency and neurodegenerative diseases like Alzheimer’s disease, Parkinson’s disease, Huntington’s disease, and multiple sclerosis [10–12].

Given that iron deficiency has been implicated in many neurodegenerative diseases, it is plausible that a similar association may be observed in psychiatric conditions. The very same biological pathways that support brain development are also involved in mood regulation and cognitive-emotional processing [13, 14]. Studies following children previously treated with IDA demonstrated poor performance in cognitive, socioemotional, and motor testing [15, 16]. As a potential consequence of these deficits, children and adolescents with IDA were also identified to be at higher risk of psychiatric disorders compared to those without any history of IDA [17]. This further supports the idea of IDA not just as a hematologic condition, but also one with robust neurodevelopmental and psychiatric outcomes. Early development of IDA in children and adolescents may also influence psychiatric vulnerability later in life.

The literature remains largely lacking and sometimes conflicting in regards to the role of iron deficiency in adult psychiatric illness. A study of Korean adults showed that anemia was significantly associated with 37% increased odds of depression in women but not significantly associated in men [18]. A nationwide database analysis study conducted in 2020 found that adult patients with IDA had a 52% increased adjusted risk of psychiatric conditions such as anxiety disorders, depression, and sleep disorders, compared to patients without IDA [19]. However, this data lacked demographic stratification to help identify at-risk adult populations, making it unclear if the risk of psychiatric conditions is uniform across sex, race, age, and other demographic variables. A major highlight of this study was the lower risk of psychiatric disorders observed in patients with IDA who had been given iron supplementation [19]. The sentiment of iron supplementation as a therapeutic intervention is not new. A growing body of evidence has shown that iron therapy can provide tangible benefits to cognition, mood, and energy even in patients without anemia [20, 21]. Specifically, patients with MDD have been shown to respond favorably to iron therapy. Children and adolescents treated with iron supplementation showed improved scores on depression scales after just 12 weeks [21]. In a population of adult patients with MDD and iron deficiency, over half of the patients experienced a reduction or even complete elimination of psychiatric symptoms [21].

Iron status is also relevant for psychosis or schizophrenia spectrum illness. Kim et al. demonstrated that latent iron deficiency (ferritin < 30 ng/mL) was associated with greater negative symptom severity [22]. More recently, a 2025 paper replicated and extended these findings in a cross-sectional analysis of first-episode patients, indicating that comprehensive iron profiles, including transferrin saturation, total iron binding capacity levels, ferritin, and serum iron, associate with negative symptoms domains including avolition and anhedonia [23]. These findings suggest that iron status may modulate central dopaminergic dysfunction and reward pathway function across diagnostic boundaries, supporting the evaluation of iron deficiency as a potentially modifiable factor in psychiatric illness.

These studies across psychiatric diagnoses provide promising support for the possibility of a modifiable link between IDA and MDD. However, little is known about how demographic factors influence the odds of MDD in patients with IDA. Understanding these factors would allow for better identification of at-risk patient populations who would derive the most clinical benefit from iron supplementation. Therefore, this study seeks to identify potential racial, ethnic, and sex-based differences in odds of major depressive disorder among patients with iron deficiency anemia. Identifying demographic variation in MDD risk among patients with IDA may improve early detection of depression and inform targeted, potentially modifiable interventions in clinical practice.

## Methods

### Data sources

This retrospective study utilized the Epic electronic health record (EHR) database from the University of California, Irvine (UCI) Medical Center. Data was accessed in a de-identified manner through the institution’s Honest Broker service, which ensures secure provision of clinical information for research. The International Classification of Disease, 10th Revision Clinical Modification (ICD-10-CM) codes were used to identify diagnoses within the database. This study was approved by the Institutional Review Board of UCI Medical Center in Orange, California (IRB No: 4289).

### Study population

**Fig. 1:**
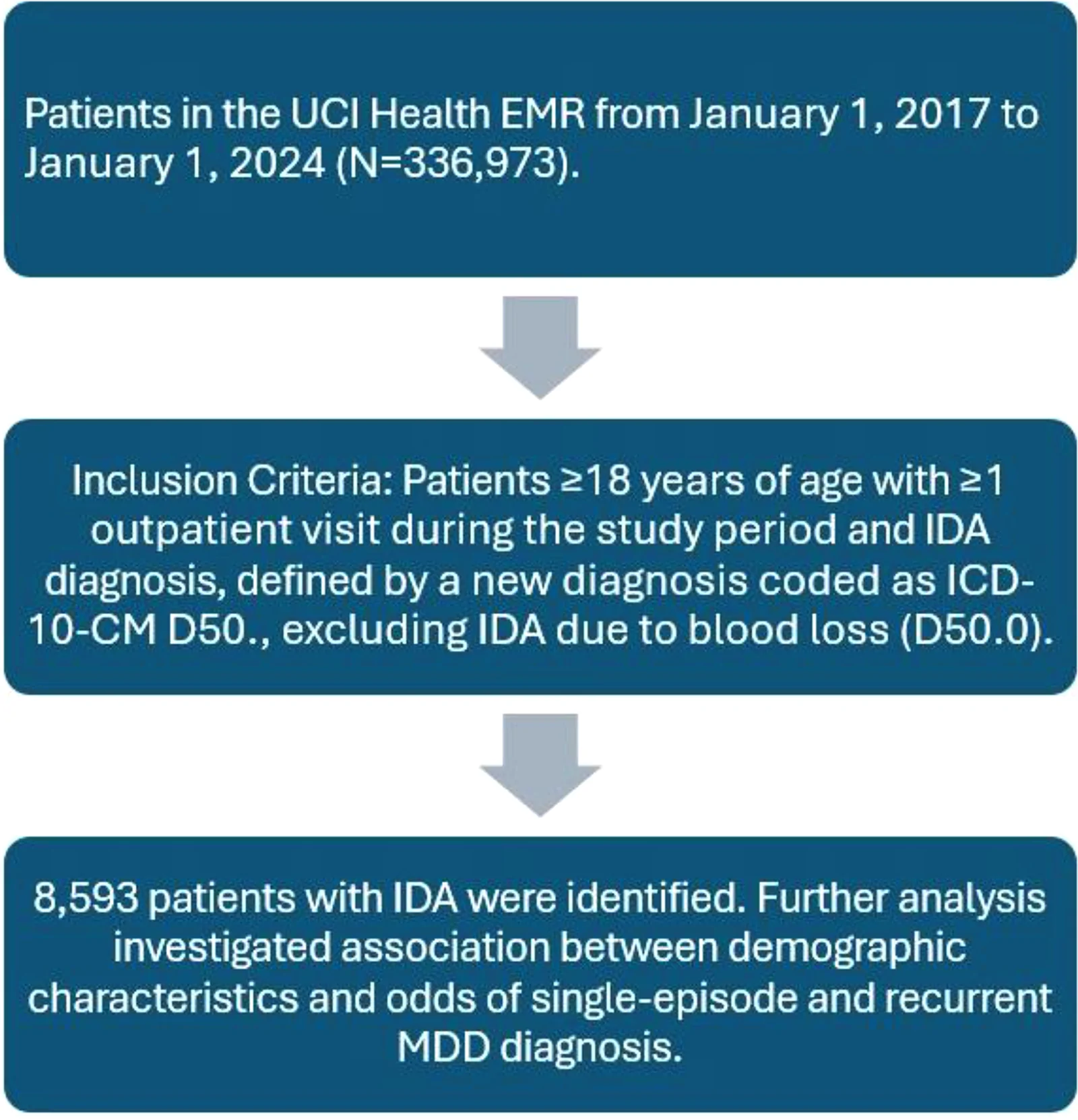
Flow diagram of cohort identification and study inclusion. Flow diagram illustrating identification of eligible patients from the electronic medical record, application of inclusion criteria, and derivation of the final analytic cohort. Abbreviations: UCI, University of California, Irvine; EMR, Electronic Medical Record; IDA, iron deficiency anemia; MDD, major depressive disorder

The study period spanned from January 1, 2017, to January 1, 2024, aligning with the implementation of the Epic electronic health record system at UCI Health. Patients aged 18 years or older as of January 1, 2017 who had at least one outpatient visit within the UCI Health system and a diagnosis of iron deficiency anemia (IDA) were included. IDA was defined by a new diagnosis coded as ICD-10-CM D50., excluding IDA due to blood loss (D50.0). Demographic variables including age, sex at birth, and ethnicity were collected. Major depressive disorder (MDD) was identified using ICD-10-CM codes for single episode (F32.*) or recurrent episodes (F33.*). MDD was classified as a single episode if the patient had only one documented depressive episode in their lifetime, and as recurrent if the patient had two or more distinct episodes separated by a period of full or near-full remission (Fig. 1).

### Statistical analysis

This analysis assessed the presence of MDD diagnoses among patients with IDA during the study period and was not designed to establish temporal or causal relationships between IDA onset and depression. Separate multivariable logistic regression models were constructed to evaluate associations between demographic characteristics and odds of single-episode or recurrent MDD diagnoses within the IDA cohort. Covariates included sex, age category, race, and ethnicity. Age was categorized into clinically relevant strata (18–24, 25–34, 35–44, 45–54, 55–64, and ≥ 65 years) to facilitate demographic interpretation and account for potential non-linear associations between age and MDD diagnoses. Reference groups were male sex, age 18–24 years, White race, and non-Hispanic/non-Latino ethnicity. Adjusted odds ratios (aORs) with 95% confidence intervals (CIs) were reported. Baseline demographic and clinical characteristics were summarized using descriptive statistics. Given the demographic nature of included covariates, substantial multicollinearity was considered unlikely and was not formally assessed. Missing race and ethnicity data were retained as “Unknown” categories within the regression models. Sparse cell counts in certain racial and ethnic subgroups were retained rather than collapsed in order to preserve demographic specificity and avoid obscuring potentially meaningful subgroup differences. Statistical significance was defined as *p* < 0.05. All analyses were performed in RStudio v4.3.2 (Boston, MA).

## Results

Among the population of 336,973 patients with at least one outpatient visit in the specified time period, 8,593 cases of IDA were identified. Among patients with IDA, 69.8% were female, 59.8% were white, and 67.4% were not Hispanic or Latino. The median age at visit was 53 years old (IQR: 37–71). Table 1 presents a summary of the full patient demographic characteristics.

**Table 1:** Demographics of IDA population

Demographics	IDA (N=8593)
**Sex**	
Female	6002 (69.8%)
Male	2591 (30.2%)
**Race**	
American Indian/Alaska Native	21 (0.2%)
Asian	1256 (14.6%)
Black or African American	326 (3.8%)
Multirace	241 (2.8%)
Native Hawaiian/Pacific Islander	50 (0.6%)
Other Race	1360 (15.8%)
Unknown	200 (2.3%)
White	5139 (59.8%)
**Ethnicity**	
Hispanic or Latino	2607 (30.3)
Not Hispanic or Latino	5793 (67.4)
Unknown	193 (2.2)
**Age**	
Age at visit, median (IQR)	53.00 [37.00, 71.00]
18–24	581 (6.8)
25–34	1202 (14.0)
35–44	1379 (16.0)
45–54	1265 (14.7)
55–64	1097 (12.8)
65+	3069 (35.7)
**MDD Diagnosis**	
Single Episode MDD	1993 (23.2)
Recurrent MDD	618 (7.2)

Abbreviations: IDA, iron deficiency anemia; MDD, major depressive disorder

Within the IDA cohort, single episode MDD was present in 23.2% of patients. Recurrent MDD was present in 7.2% of patients. Multivariable logistic regression models evaluated associations between demographic characteristics and odds of single-episode or recurrent MDD among patients with IDA. Reference groups were male sex, age 18–24 years, White race, and non-Hispanic/non-Latino ethnicity.

**Fig. 2:**
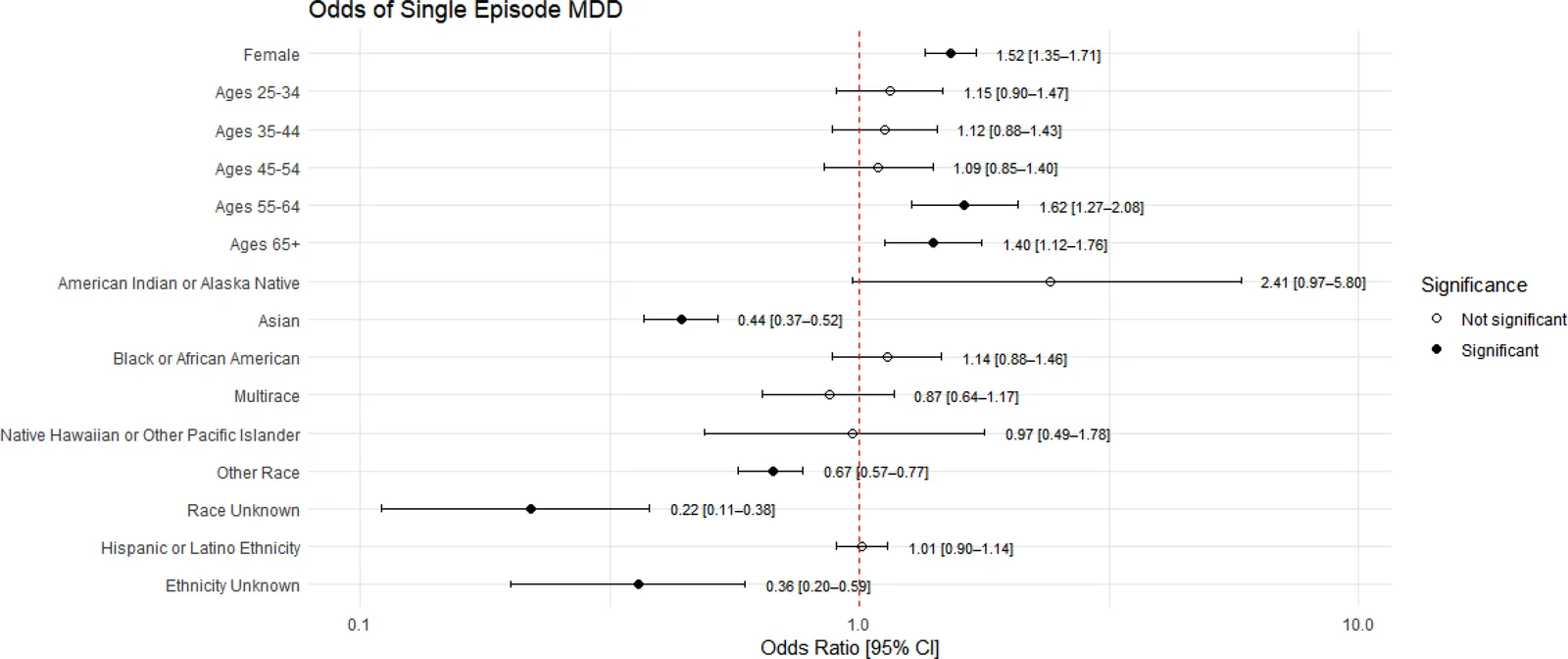
Adjusted odds ratios for single-episode major depressive disorder (MDD) diagnoses among patients with iron deficiency anemia (IDA). Forest plot displaying adjusted odds ratios and 95% confidence intervals derived from multivariable logistic regression models evaluating demographic associations with documented single-episode MDD diagnoses within the IDA cohort. Reference groups were male sex, age 18–24 years, White race, and non-Hispanic/non-Latino ethnicity

Among patients with IDA, females had higher odds of single episode MDD (Odds Ratio (OR) 1.52, 95% CI [1.35–1.72], *p* < 0.001) and recurrent MDD (OR 1.84, 95% CI [1.50–2.28], *p* < 0.001) relative to males. Compared to patients ages 18–24, patients ages 25–34, 35–44, and 45–54 did not indicate significantly different odds of either single episode or recurrent MDD (all *p* > 0.05). However, patients ages 55–64 had greater odds of single episode MDD (OR 1.62, 95% CI [1.27–2.08], *p* < 0.001) but not recurrent MDD (OR 1.03, 95% CI [0.70–1.54], *p* = 0.88). Similarly, patients ages 65 + demonstrated greater odds of single episode MDD (OR 1.40, 95% CI [1.12–1.76], *p* = 0.003), though there was no significant association found for recurrent MDD (OR 1.09, 95% CI [0.78–1.57], *p* = 0.62). Adjusted associations for single-episode MDD are illustrated in Fig. 2.

Relative to White patients, Asian individuals had significantly decreased odds of single episode (OR 0.44, 95% CI [0.37–0.52], *p* < 0.001) and recurrent MDD (OR 0.39, 95% CI [0.28–0.52], *p* < 0.001). Patients who identified as “Other Race” also exhibited significantly lower odds of single episode MDD (OR 0.67, 95% CI [0.57–0.77], *p* < 0.001) and recurrent MDD (OR 0.49, 95% CI [0.37–0.65], *p* < 0.001). Patients of unknown race were found to have the lowest odds of single episode MDD (OR 0.22, 95% CI [0.11–0.38], *p* < 0.001) and recurrent MDD (OR 0.12, 95% CI [0.02–0.39], *p* = 0.004) compared to white patients. Patients who identified as “Multirace” or Native Hawaiian or Pacific Islander did not demonstrate significantly different odds in either single episode or recurrent MDD. Black or African patients showed a slightly higher odds of single MDD but not of significance. American Indian or Alaska Native individuals demonstrated a non-significant trend toward higher odds of single episode MDD (OR 2.41, 95% CI [0.97–5.80], *p* = 0.051), but no significant difference was observed for recurrent MDD.

**Fig. 3:**
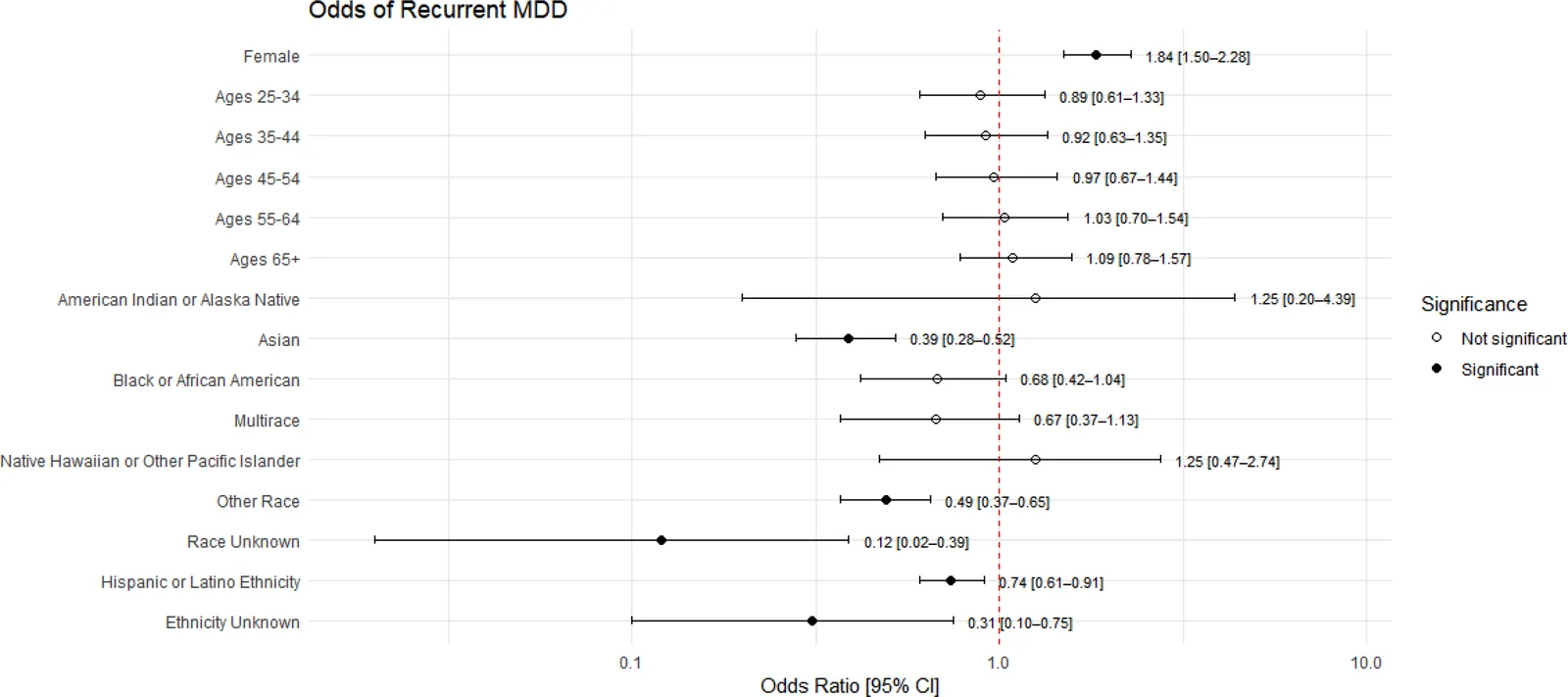
Adjusted odds ratios for recurrent major depressive disorder (MDD) diagnoses among patients with iron deficiency anemia (IDA). Forest plot displaying adjusted odds ratios and 95% confidence intervals derived from multivariable logistic regression models evaluating demographic associations with documented recurrent MDD diagnoses within the IDA cohort. Reference groups were male sex, age 18–24 years, White race, and non-Hispanic/non-Latino ethnicity

Hispanic or Latino patients had significantly lower odds of recurrent MDD (OR 0.74, 95% CI [0.61–0.91], *p* = 0.004) compared to patients who were not Hispanic or Latino, but no significant difference was observed for single episode MDD. Patients of unknown ethnicity demonstrated significantly lower odds of both single episode (OR 0.36, 95% CI [0.20–0.59], *p* < 0.001) and recurrent MDD (OR 0.31, 95% CI [0.10–0.75], *p* = 0.024). A summary of these associations is presented in Table 2, and the adjusted associations for recurrent MDD are shown in Fig. 3.

**Table 2:** Multivariable logistic regression analysis of demographic associations with single-episode and recurrent MDD diagnoses among patients With IDA

Group	Single Episode MDD			Recurrent MDD		
	OR	CI (95%)	*P* value	OR	CI (95%)	*P* value
**Male**	**Reference**	-	-	**Reference**	-	-
Female	1.518	(1.345–1.715)	**< 0.001**	1.843	(1.500-2.278)	**< 0.001**
**Age 18–24**	**Reference**	**-**	**-**	**Reference**	**-**	**-**
Age 25–34	1.146	(0.899–1.467)	0.275	0.894	(0.610–1.326)	0.569
Age 35–44	1.117	(0.880–1.426)	0.367	0.920	(0.634–1.355)	0.667
Age 45–54	1.089	(0.854–1.396)	0.495	0.973	(0.668–1.438)	0.889
Age 55–64	1.619	(1.267–2.080)	**< 0.001**	1.032	(0.699–1.542)	0.875
Age 65+	1.401	(1.121–1.761)	**0.003**	1.094	(0.778–1.57)	0.616
**White**	**Reference**	**-**	**-**	**Reference**	**-**	**-**
Black or African	1.14	(0.881–1.463)	0.312	0.678	(0.421–1.039)	0.091
Asian	0.438	(0.366–0.523)	**< 0.001**	0.387	(0.282–0.521)	**< 0.001**
Native Hawaiian or Pacific Islander	0.967	(0.492–1.785)	0.919	1.249	(0.473–2.743)	0.614
American Indian or Alaska Native	2.406	(0.966–5.801)	0.051	1.247	(0.197–4.393)	0.769
Multirace	0.869	(0.635–1.171)	0.367	0.672	(0.370–1.126)	0.159
Other Race	0.665	(0.571–0.773)	**< 0.001**	0.492	(0.368–0.647)	**< 0.001**
Race Unknown	0.219	(0.114–0.380)	**< 0.001**	0.124	(0.020–0.393)	**0.0036**
**Not Hispanic or Latino**	**Reference**	**-**	**-**	**Reference**	**-**	**-**
Hispanic or Latino	1.013	(0.898–1.144)	0.828	0.741	(0.605–0.905)	**0.0035**
Ethnicity Unknown	0.358	(0.203–0.590)	**< 0.001**	0.313	(0.095–0.753)	**0.0236**

Abbreviations: IDA, iron deficiency anemia; MDD, major depressive disorder; OR, odds ratio; CI, confidence interval. Reference groups were male sex, age 18–24 years, White race, and non-Hispanic/non-Latino ethnicity

## Discussion

In this large, real-world cohort, iron deficiency anemia identified a population with a high burden of major depressive disorder, with meaningful variation across demographic subgroups. Nearly one-quarter of patients with IDA were diagnosed with single-episode MDD, and over 7% with recurrent MDD, underscoring the relevance of psychiatric risk assessment in patients with chronic medical conditions.

Given the critical role of iron in neurotransmitter synthesis, energy metabolism, and brain function, the overall observed trend of elevated risk for MDD in patients with IDA, consistent across all subgroups, is consistent with emerging evidence that iron deficiency has neurobiological effects that may contribute to depression. Dysregulation of hippocampal glucocorticoid receptor signaling has been proposed as a potential mechanism, as cerebral iron deficiency in animal models leads to neuronal injury, reduced neurogenesis, impaired HPA axis negative feedback, and exacerbation of depressive-like behaviors [24]. Additionally, a 2008 study based on rodent models demonstrated that reduced brain iron leads to impaired dopamine D2 receptor function in the striatum, increased endogenous opioid activity, and disrupted ferritin-dependent processes in the hippocampus with corresponding deficits in learning and memory. These findings provide a mechanistic framework for understanding iron deficiency’s link to depressive symptomatology: depressive cognitive schemas may partly reflect iron-dependent constraints on reward processing, neuroplasticity, and neuronal injury rather than purely psychological processes [25]. While these mechanistic models provide biological plausibility, the present findings primarily support IDA as a clinically relevant risk marker rather than a confirmed causal factor for depression.

### Sex differences

Female patients with IDA demonstrated a substantially higher risk of both single and recurrent MDD compared to male patients with IDA. These sex differences are consistent with prior literature demonstrating an association between anemia and depressive symptoms in women of reproductive age including a meta-analysis that found that anemia was associated with a 53% increased risk of maternal depression that was consistent across antepartum and postpartum periods [26]. Additionally, a 2025 study of adolescents with iron deficiency without anemia showed that lower striatal iron content was associated with greater depressive symptoms and more impairment with more pronounced effects in females [27]. At baseline, female patients are known to have a greater prevalence of iron deficiency due to blood loss during menstruation or pregnancy. The observed increase in both single and recurrent MDD demonstrates that iron may play a role in both the initial odds of a depressive episode along with continued persistent depression or relapse of depression. However, causal inference cannot be established due to the observational nature of this study. Such differences may be due to a combination of biological, psychosocial, and healthcare systems-level influences.

### Age differences

Older adults (≥ 55 years) were observed to have an increased risk of single MDD but no significant difference in risk for recurrent MDD. This finding may demonstrate an elevated vulnerability to the first odds of depression, although the mechanisms remain unclear. Prior studies have theorized that functional decline, medical comorbidities, and metabolic and neurological dysfunction due to inflammatory processes in aging may contribute to the risk of late-onset depression in older adults [28–31]. The lack of association with recurrent MDD in older adults suggests that late-onset odds of a first MDD diagnosis may represent a distinct phenotype of depression associated with age-related neurodegenerative or vascular changes that may not be present with recurrent depression [32]. Younger and middle-aged adults were not observed to have elevated risk of single or recurrent MDD compared to the reference group of patients aged 18–24 years old. Thus, age-related risks associated with IDA may appear more prominently only later in life.

### Race and ethnicity differences

Asian patients with IDA demonstrated substantially lower odds of both single and recurrent MDD compared to other racial and ethnic groups. While these findings may reflect biological mechanisms, they may also be influenced by disparities in diagnosis or access to mental health services. Past studies have demonstrated a four to ten times greater risk of lifetime depression in Western countries compared to Asia, with this discrepancy largely associated with cultural differences including emotional regulation strategies and stigma towards mental health disorders [33, 34]. Asian Americans are also less likely to express specific somatic and psychological symptoms of depression than European Americans even after matching for depression severities, thereby potentially contributing to disparities in diagnostic threshold for depression [35]. 

Patients who identified as Hispanic or Latino demonstrated lower odds of recurrent MDD but no significant difference in odds of single MDD. Thus, differences may reflect disparities in access to mental health treatment or desire for continued follow-up as prior studies have demonstrated a lower rate of perceived need for treatment despite increased odds of unmet needs [36]. These findings should be interpreted in the context of known underdiagnosis and differential help-seeking behaviors across racial and ethnic groups, which may contribute to observed differences in documented MDD prevalence. Still, such findings highlight the need for increased accessibility of culturally competent mental health services.

Notably, Black or African American and American Indian or Alaska Native patients did not demonstrate statistically significant differences in MDD incidence compared to White patients. Several racial subgroup analyses should be interpreted cautiously due to small sample sizes in certain categories, particularly among American Indian/Alaska Native and Native Hawaiian/Pacific Islander patients. These sparse subgroup counts may have resulted in unstable odds ratio estimates and reduced precision, as reflected by wider confidence intervals. Although categories were retained separately to preserve demographic specificity, future studies with larger subgroup representation or alternative modeling approaches for sparse data are needed to better characterize these associations.

### Strengths and limitations

One of the primary strengths of our study is the ability to represent a large portion of California’s Orange County, a portion of which had been previously unstudied for psychiatric illness in the context of IDA. UCI Health’s facilities extend throughout the county, and UCI Health serves a large population due to the lack of a county psychiatric hospital in the region. A dataset of over 8,500 patients allowed fairly robust estimation of psychiatric outcomes, improved statistical power to detect associations, and enhanced generalizability to a diverse, real-world population. Some important limitations for this study include the lifestyle information that is not sufficiently documented for each patient or patient visit in the health record such as sleep quality/quantity, dietary habits, or exercise frequency, which may be confounding variables.

Furthermore, in identifying cases of IDA, the use of ICD-10 codes does not necessarily represent a laboratory-confirmed diagnosis of IDA but rather a clinical diagnosis. In conjunction with the exclusion of the D50.0 code to avoid cases of acute blood loss, there is a potential risk of missing clinically significant cases due to physician coding errors in which an acute blood loss was attributed to the diagnosis despite an underlying IDA. However, this risk is outweighed by the benefits of being able to more clearly isolate cases of IDA caused by nutritional deficiencies or chronic conditions. Of note, due to inconsistency in the data coded in the EHR for patients with IDA, labs indicating IDA severity, IDA treatment status, MDD severity measures, and time data elucidating a temporal relationship between IDA and MDD could not be included, Thus, the findings should be interpreted with caution due to these potential confounders. Additionally, as with many studies, the true prevalence of MDD was most likely underestimated as individuals who did not seek psychiatric care are not included in the EHR. Residual confounding due to unmeasured socioeconomic factors, health literacy, and access to mental health services may also influence observed associations.

Our results indicate a robust statistical association between iron deficiency anemia and the presence of major depressive disorders, consistent across most demographic subgroups. The extremely low p-values observed in chi-square analyses suggest that this relationship is unlikely to be due to chance alone. However, given our large sample size, statistical significance may reflect even modest absolute differences, and effect sizes should be interpreted with caution.

### Clinical implications

This cohort study identified significant demographic variation in the likelihood of MDD diagnosis among patients with IDA. These findings underscore the importance of considering demographic context in clinical psychiatric evaluation and support future investigation into the mechanisms underlying these observed differences. Improved understanding of demographic-specific risk patterns may also inform targeted screening, prevention, and treatment strategies, thereby enhancing population-level mental health outcomes. Further study is required to investigate the relationship between the severity of IDA and the severity of MDD and/or presence of psychotic features as well as to better control for confounding factors such as lifestyle factors or family history of mental illness. Additional study may also focus on the changes in MDD prevalence and/or severity with sufficient treatment of IDA.

## Conclusion

In this large, real-world cohort, IDA identifies a medically vulnerable population with a substantial burden of both single-episode and recurrent MDD, with important demographic variation. Nearly 25% of patients with IDA experienced a single depressive episode and over 7% of recurrent MDD, highlighting the clinical significance of psychiatric comorbidity in this population. Older age and female sex were associated with higher odds of MDD among patients with IDA, while Asian race was associated with lower odds. Although causal inference cannot be established due to the observational design, these findings are plausible given iron’s role in neurochemical and neuroendocrine function. Taken together, the results support the clinical relevance of iron deficiency in psychiatric assessment and underscore the potential value of integrated screening for depressive symptoms in patients with IDA. Future longitudinal and interventional studies are needed to clarify temporal relationships and determine whether treatment of IDA mitigates depression risk or recurrence.

## Data Availability

The datasets analyzed during the current study are not publicly available because they contain protected health information and are subject to institutional and ethical restrictions. De-identified data is available through the online data repository as follows.Hardoy, Christian (2026), “Demographic Predictors of Major Depressive Disorder Among Patients with Iron Deficiency Anemia: A Retrospective Cohort Study”, Mendeley Data, V1, https://doi.org/10.17632/www4mpy6sb.1.

## Abbreviations

IDA: Iron deficiency anemia
MDD: Major depressive disorder
EHR: Electronic health record
UCI: University of California, Irvine
ICD-1-CM: The International Classification of Disease, 10th Revision Clinical Modification
aOR: Adjusted odds ratio
CI: Confidence interval
